# Clustering trunk movements of children and adolescents with neurological gait disorders undergoing robot-assisted gait therapy: the functional ability determines if actuated pelvis movements are clinically useful

**DOI:** 10.1186/s12984-023-01200-0

**Published:** 2023-06-03

**Authors:** Florian van Dellen, Tabea Aurich-Schuler, Nikolas Hesse, Rob Labruyère

**Affiliations:** 1 Swiss Children’s Rehab, Children’s University Hospital Zurich, Mühlebergstrasse 104, 8910 Affoltern Am Albis, Switzerland; 2 Sensory-Motor Systems Lab, Department of Health Sciences and Technology, ETH Zurich, Tannenstrasse 1, 8092 Zurich, Switzerland; 3 Children’s Research Center, Children’s University Hospital Zurich, University of Zurich, Steinwiesstrasse 75, 8032 Zurich, Switzerland

**Keywords:** Center of mass displacement, Weight shifting, Pelvis movements, Rehabilitation, Marker-less motion tracking system, Trunk control, Balance

## Abstract

**Introduction:**

Robot-assisted gait therapy is frequently used for gait therapy in children and adolescents but has been shown to limit the physiological excursions of the trunk and pelvis. Actuated pelvis movements might support more physiological trunk patterns during robot-assisted training. However, not every patient is expected to react identically to actuated pelvis movements. Therefore, the aim of the present study was to identify different trunk movement patterns with and without actuated pelvis movements and compare them based on their similarity to the physiological gait pattern.

**Methods and results:**

A clustering algorithm was used to separate pediatric patients into three groups based on different kinematic reactions of the trunk to walking with and without actuated pelvis movements. The three clusters included 9, 11 and 15 patients and showed weak to strong correlations with physiological treadmill gait. The groups also statistically differed in clinical assessment scores, which were consistent with the strength of the correlations. Patients with a higher gait capacity reacted with more physiological trunk movements to actuated pelvis movements.

**Conclusion:**

Actuated pelvis movements do not lead to physiological trunk movements in patients with a poor trunk control, while patients with better walking functions can show physiological trunk movements. Therapists should carefully consider for whom and why they decide to include actuated pelvis movements in their therapy plan.

**Supplementary Information:**

The online version contains supplementary material available at 10.1186/s12984-023-01200-0.

## Background

The improvement of gait function is a frequent rehabilitation goal in pediatric rehabilitation [1]. Besides traditional physiotherapeutic interventions, robot-assisted gait therapies (RAGT) have become popular, including the Lokomat (Hocoma AG, Volketswil, Switzerland). These robots are a promising tool for therapists because they can provide a higher training intensity compared to conventional therapies [2]. Several studies in children and adults have shown that RAGT can induce improved strength, endurance, and kinematics and is similarly effective to conventional physiotherapy [2–6]. However, although several studies investigated effectiveness in pediatrics, most of them did not yield conclusive results. Problems especially arise from the low case numbers and the high variability of the patients’ pathologies and ages [7]. Accordingly, recent work has focused more on optimally using the device by analyzing the immediate physiological effects of different features that the robotic interventions offer [8–11].

**Fig. 1:**
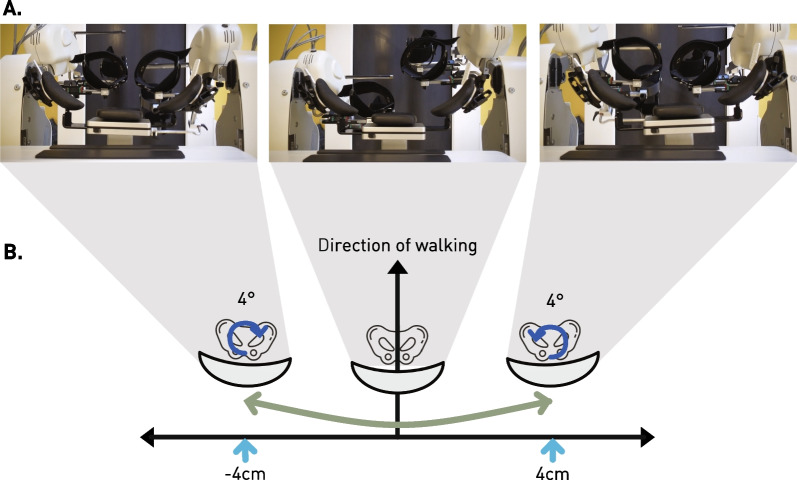
Working Principle of the FreeD Module: **A** The images show the FreeD module from the top view in different positions. **B** The FreeD module moves the pelvis on a combined mediolateral translation (light blue) and axial rotation (dark blue). Maximum values are 4 cm per side and 4 degrees axial rotation. The combined movement path of the pelvis is shown in light green

Traditionally, the Lokomat fixates the pelvis and restricts the mediolateral movements. However, mediolateral shifts of the center of mass towards the stance leg are a crucial component of stable gait [12]. In physiological gait, this is achieved with a sinusoidal trajectory of the pelvis, while the thorax is kept relatively stable above it [13–15]. Restricting mediolateral movements leads to compensatory trunk movements [16, 17] and can alter muscle activation patterns [18], suggesting that shifting the pelvis and trunk should also be considered in gait rehabilitation. Therefore, the FreeD module was added to the Lokomat in 2014 [9]. The FreeD module (Fig. 1) is a hard- and software extension available for the LokomatPro. It consists of a pelvis support shell that is attached to the patient with textile straps. It actively controls the weight shifting of patients walking in the Lokomat with a maximal lateral excursion of 4 cm per side. The mediolateral movement is superposed with a maximal axial rotation of ± 4 degrees (Fig. 1B). This leads to a pelvis movement on a semi-elliptical path (light green arrow, Fig. 1). The magnitude of the lateral excursion can be adjusted in the software of the Lokomat. The lateral excursion is timed so that it peaks around the midstance phase of the ipsilateral stance leg and is synchronized with gait speed. The timing of the movement relative to the gait cycle can also be adjusted in the software, but needs to be previously activated in the system settings. Furthermore, the upper and middle cuffs attaching the legs can be released which allows the legs to follow the pelvic movement.

However, the impact of such actuated movements on the frontal plane kinematics are unclear. The manufacturer claims that the FreeD module allows the patients to naturally shift their weight over the standing leg, to activate core muscles and to “experience balance aspects”. A proof-of-concept study showed that the FreeD module allows a more natural lateral trunk movement (pelvis and thorax) and promotes muscle activation patterns similar to normal treadmill walking in healthy adults [16]. In contrast, children and adolescents with neurological gait disorders showed less physiological leg muscle activation patterns but substantial variability [9]. Two considerations could help to explain these findings: (1) While healthy participants actively control the pelvic shift introduced by the FreeD module, patients might be passively moved. Many children with neurological gait disorders have deficits in trunk control ability [11] and the actuated pelvis movement might rather disturb them than support a physiological movement. (2) The studies performed so far include neurological patients with very different forms of gait impairments [9]. This heterogeneity was not considered in the analyses, but mixing data from a diverse population could be detrimental and obscure contradicting reactions of subgroups.

Consequently, in order to assess the clinical utility of such actuated movements, it is crucial to understand the variety of behaviors that patients may exhibit. The aim of the present study was therefore to identify different trunk movement patterns with and without actuated pelvic movements, to compare them on the basis of their similarity to physiological gait patterns, and to relate them to the patients' functional capacity. We hypothesized to find 3 distinct groups of patients: (1) An unstable group, with an unstable trunk reaction to the fixed pelvis condition, as well as to the actuated pelvis condition. (2) A partially stable group, with a stable trunk reaction to the fixed pelvis condition, but with an unstable trunk reaction to the actuated pelvis condition. (3) A stable group with a stable trunk reaction to both conditions in line with the findings in healthy adults [16]. We expected that the results of clinical assessments for trunk control, balance, and walking performance would differ between the groups.

## Methods

### Participants

Five to twenty years old in- and outpatients with a neurological gait disorder were recruited at the Swiss Children’s Rehab in Affoltern am Albis, Switzerland, by convenience sampling. Patients were excluded if they could not communicate pain and discomfort, did not understand simple instructions, or did not fulfil the requirements for Lokomat usage described in the Lokomat’s handbook [19]. Furthermore, three completed Lokomat therapies before the measurement were required as familiarization. The research project was performed in accordance with the Declaration of Helsinki, and the cantonal ethics committee of the Canton Zurich, Switzerland, approved the project (BASEC-Nr: 2019-02116). All participants and/or their legal guardians signed an informed consent form. The recruitment took place between December 2019 and April 2021.

### Study protocol

All participants attended a block of walking conditions in the LokomatPro Version 6 (Hocoma, Volketswil, Switzerland) with a total walking time of 20 min. A trained therapist adjusted the settings such as joint range of motions, etc., individually to each patient. Patients were instructed to loosely place their arms on the parallel bars with the elbows flexed at 90 degrees. Standardized Instructions were kept to a minimum. A list of all possible instructions can be found in the Additional file 1: Supplementary Material A. The bodyweight support (max. 30% of body weight) was reduced as much as possible while still ensuring proper knee extension during the stance phase. Robotic guidance was set to 100%. The walking speed was gradually increased until the participants perceived it as comfortable. In the first 10 min, the participants familiarized themselves with the movements of the robotic orthoses. Then, the participants walked for 10 min with two different settings of the FreeD module, whereby the order was randomized. Each condition lasted 5 min. In one condition, the pelvis support position was fixed (subsequently referred to as “*FreeD Off”*), while in the other condition, the FreeD module was activated such that the pelvis support module described a lateral excursion of 2 cm per side (referred to as *“FreeD On”*). In addition, the cuffs at the thigh and upper shank were released to allow a mediolateral shift in the *FreeD On* condition only.

### Outcomes

**Fig. 2:**
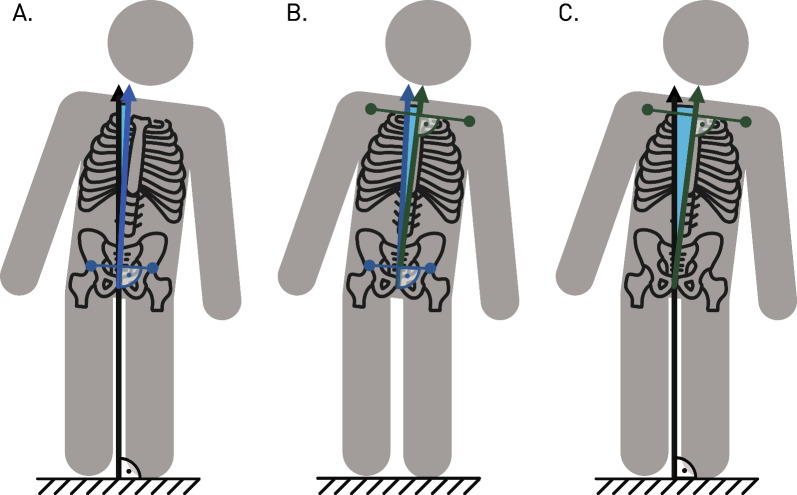
Visual description of trunk obliquities: To evaluate the kinematic behavior in the frontal plane, 3 axes were defined. The thorax axis by connecting the two shoulder joints (in green), the pelvis axis by connecting the two hip joints (in blue) and an axis parallel to gravity (in black). From these axes, Pelvis to World obliquity (**A**), Thorax to Pelvis obliquity (**B**) and Thorax to World obliquity (**C**) were calculated

The primary outcome measure was the kinematic pattern of the patients. They were recorded at 30 Hz with an Azure Kinect DK RGB-D camera (Microsoft, Seattle, USA) placed directly in front of the patients to minimize occlusions by the Lokomat. The recordings were transformed offline into kinematic data using a custom 3D body tracking method, which fits a virtual body model [20] to the recorded point clouds of the Azure Kinect DK [21]. The model provides kinematic data as three-dimensional time series for 24 joint positions and angles. As thorax and pelvis stability in the frontal plane was expected to be most affected by the mediolateral shifts, Pelvis to World, Thorax to Pelvis, and Thorax to World obliquities were calculated based on the hip and shoulder joints (Fig. 2). The resulting time series were then segmented into individual strides and time normalized (0–100% of the gait cycle). Left heel strikes were identified as the most anterior position of the left ankle joint in the sagittal plane [22]. To reduce carry-over effects between the conditions, only the mean of the last 25 strides of each condition was included in the analysis. In addition, reference data from 10 typically developing children and adolescents during treadmill walking were used from a different study [21] and processed with the same approach.

To evaluate trunk control and walking ability, 3 different assessments [Trunk Control Measurement Scale (TCMS), Gillette Functional Assessment Questionnaire (GFAQ) and modified Timed up and Go Test (TUG)] were performed before walking in the Lokomat as secondary outcome measures. (1) The TCMS [23] evaluates trunk control. It sums a maximum of 58 points in three different domains, namely static sitting balance (20 points), selective movement control (28 points), and dynamic reaching (10 points). (2) The GFAQ obtains a measure of the walking behavior in daily life [24, 25]. (3) The TUG assesses functional balance during walking [26, 27]. Participants used their usual walking aids for the TUG and conducted the test twice with the instruction to do the test as fast as possible without running after a “3, 2, 1, go!”-signal. The faster of the two trials counted. As 6 patients could not perform this test, we did not include the results of the Timed Up and Go Test in the statistical analysis.

### Clustering and statistical analyses

All statistical analyses were performed with RStudio (RStudio Team (2015). RStudio: Integrated Development for R. RStudio, Inc., Boston, MA, USA, http://www.rstudio.com).

In the past, subgroups (clusters) of children with cerebral palsy that differed by their gait pattern have been successfully identified with clustering [28]. As trunk movements are continuous, no hard borders between the clusters can be defined. Therefore, fuzzy clustering was used, which allows for a probabilistic cluster assignment [29]. Typical gait parameters like maximum/minimum angles or range of motion are unrelated to the timing in the gait cycle, which also contains essential information. Therefore, a whole gait cycle approach was used based on a dynamic time-warping distance [30]. Consequently, the participants were separated into 3 clusters (motivated by our hypotheses) with a fuzzy c-means clustering based on dynamic time-warping distances, as implemented in the *dtwclust* package [31]. To test whether the resulting clusters agree with our hypotheses, the mean trajectories of each cluster were correlated to the reference trajectories of the trunk from treadmill walking using Pearson’s correlation coefficient. Correlation coefficients were interpreted as |r|< 0.20, “very weak”; 0.20–0.39, “weak”; 0.40–0.59, “moderate”; 0.60–0.79, “strong” and 0.80–1.00 “very strong relationship” [32]. Strong and very strong positive correlations were considered as stable trunk patterns. The relative movement between pelvis and thorax was the main criterion for the group labels.

Furthermore, we tested the clusters for differences in functional capacity evaluated by the clinical assessments with a multivariate analysis of variance. The assumption of homogeneity of variances was violated. Therefore, we performed a non-parametric MANOVA with TCMS and GFAQ as dependent variables and the assigned cluster as the independent variable. The alpha level was defined at 0.05. Post-hoc analyses were performed with Kruskal–Wallis tests for each dependent variable individually.

## Results

**Fig. 3:**
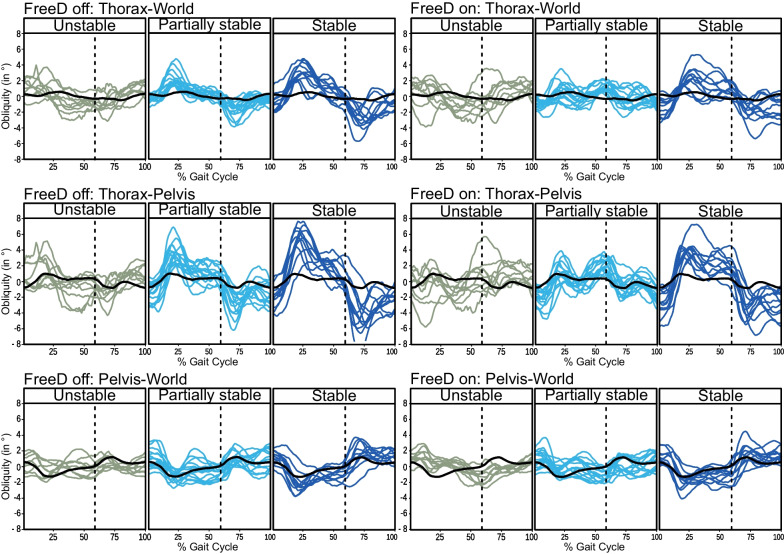
Kinematic response of the patients per cluster: The participants were assigned to 3 clusters. Each line represents the mean of the respective angle over the gait cycle beginning with the left heel strike of a single subject. The black line represents the reference trajectory from children and adolescent treadmill gait. The clusters were labelled based on how well they correlated with the reference data. A positive angle indicates that the right (contralateral) joint is higher than the left (ipsilateral) joint. The dashed line marks the approximate transition from stance to swing phase of the left leg

Thirty-five out of 38 participants were included in the analysis. A table containing the patient characteristics and information on their bodyweight support and gait speed during the recording can be found in Additional file 2: Supplementary Material B. Two participants had to be excluded due to malfunctioning of the measurement equipment and one participant due to poor compliance. The clustering resulted in cluster sizes of 9, 11 and, 15 patients. Their frontal plane kinematic patterns of the pelvis and thorax are depicted in Fig. 3.

The correlations between the cluster patterns and the reference data agreed well with our hypotheses (Table 1). Negative correlations of the thorax were found for the cluster with 9 participants, which was consequently labeled as unstable. The cluster with 15 participants had moderate to strong correlations of the thorax in the *FreeD Off* condition but only very weak to moderate correlations in the *FreeD On* condition and was therefore labeled as partially stable. Moderate to very strong correlations of the thorax were found for the cluster with 11 participants, which hence was labeled as stable.

**Table 1:** Pearson Correlation Coefficients between mean cluster trajectory and reference

	FreeD Off			FreeD On		
	Pelvis-World	Thorax- Pelvis	Thorax-World	Pelvis-World	Thorax- Pelvis	Thorax-World
Unstable	− 0.62	− 0.17	0.16	− 0.4	− 0.31	− 0.71
Partially Stable	0.01	0.55	0.76	− 0.16	0.41	0.06
Stable	0.79	0.84	0.66	0.83	0.81	0.47

**Fig. 4:**
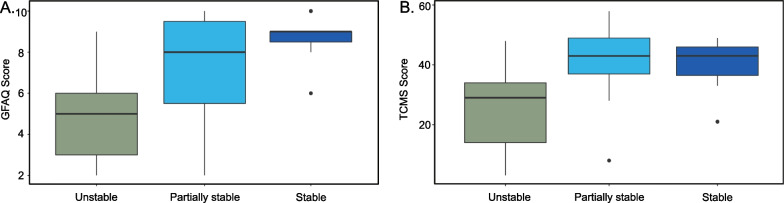
Boxplots of clinical assessment scores per cluster: The identified clusters significantly differed in both **A** Gillette Functional Assessment Questionnaire (GFAQ, maximum 10 points). **B** Trunk Control Measurement Scale (TCMS, maximum 58 points). Especially the unstable cluster showed lower scores than the other two. The partially stable cluster had a slightly lower GFAQ score

The cluster allocation was reflected in the clinical scores. The cluster had a significant effect on the clinical scores (non-parametric MANOVA: F = 9.55, df = 3, p = 0.048). Post-hoc analyses revealed clusters significantly differed in both the TCMS score (Kruskal–Wallis: Chi-Square = 6.205, df = 2, p = 0.045) and the GFAQ (Kruskal–Wallis: Chi-Square = 9.125 m df = 2, p = 0.010). Especially the unstable participants exhibited lower TCMS and GFAQ scores than the other two groups (see Fig. 4). The partially stable and stable participants had very similar average TCMS scores of 41 and 40 points, respectively. The partially stable participants tended towards a lower average GFAQ score than the stable participants, with a median of 8 and 9 points, respectively, but showed a higher variability.

## Discussion

It was found that pediatric patients react variably with their trunk to walking with a fixed pelvis or with guided pelvis movements during robot-assisted gait therapy (RAGT). These differences were also reflected in clinical assessment scores. Especially the participants with an unstable thorax had poorer trunk control and walking performance than the other participants.

### Children and adolescents with a low gait performance have difficulty to stabilize their trunk

Participants that were assigned to the unstable cluster had poorer trunk control and gait performance than those assigned to the other two clusters. This observation aligns well with the clinical experience, that many patients participating in RAGT have difficulties keeping their trunk upright. As these participants already have difficulties keeping their trunk upright with a fixed pelvis support, it is no surprise that the situation does not improve by activating the pelvis support. Likely, participants with poor trunk control are passively pushed around and struggle with the additional kinematic freedom. In contrast, two participants with a poor trunk control were found in other clusters (outliers in Fig. 4). This observation would not agree with this interpretation, but a closer look at the video footage and the test results suggests that these patients might have selected a movement strategy in the Lokomat with a strong co-contraction of trunk muscles, which led to a rigid behavior. This behavior led to a similar pattern to other clusters despite the difficulty of this patients with selective trunk movements revealed in the TCMS. Nevertheless, the interdependence of trunk control and walking function has been well established for different conditions, including cerebral palsy, stroke and spinal cord injury [23, 33, 34]. Considering this interdependence, trunk behavior should be accounted for when choosing an optimal RAGT setup, especially in patients with severely reduced trunk control. Since it was found that Lokomat therapy can improve frontal plane kinematics during walking even with a fixed pelvis [35], improving trunk control does not necessarily require a lateral displacement of the pelvis, as performed by the FreeD module. In addition, children with cerebral palsy can exhibit less physiological muscle activation patterns when walking with the FreeD module switched on [11]. A reduced task complexity might help these patients to better focus on their trunk stability while walking and even increase retention of the learned task [36]. In patients with low trunk control ability, it might therefore make sense to train trunk control without actuated pelvis movements.

### Better-performing patients behave similarly to healthy adults

At the other end of the spectrum, high correlations with treadmill gait were found in both conditions, indicating that these patients stabilize their thorax well. The kinematic pattern found is marked by a slightly elevated contralateral hip joint during the stance phase to support the foot clearance of the swing leg (Fig. 3). At the same time, the thorax is slightly tilted towards the swing leg, which probably stabilizes the center of mass and elongates the contralateral side of the trunk. This is in line with findings in healthy adults [37]. In the *FreeD On* condition, peaks of the trunk obliquity were slightly reduced. This could indicate that the FreeD module’s movement reduced the compensatory patterns seen with a fixed pelvis support in this group, which would be in line with the findings for healthy adults [16, 38]. Clinically, this would support the use of the FreeD module in this patient group with relatively high gait performance and trunk control ability. Therefore, therapists should consider whether the additional pelvis movement can support their therapy goal. However, we would like to emphasize that choosing a relevant therapy goal, especially in this group, is essential, as the benefits of RAGT for patients with a high gait performance are disputed [39].

### Actuated pelvis movements can disturb thorax stability

The movement patterns of the partially stable cluster share characteristics with both the stable and the unstable cluster. While the partially stable cluster behaved similarly to the stable cluster in the *FreeD Off* condition, the Thorax to World and Thorax to Pelvis obliquities are marked by an additional peak around toe-off (dashed line in Fig. 4) in the *FreeD On* condition and correlate less with the physiological pattern. As the participants in the partially stable cluster had a high trunk control ability, they might be able to stabilize their thorax well in the absence of actuated pelvis movements. In contrast, the trajectory of the guided pelvis module might not exactly comply with the patients’ physiological trajectory and therefore disturb their gait and induce the third peak. Most patients in this group were marked by a lower gait performance than the stable group (mean GFAQ of 7.4 instead of 8.5). While patients with a GFAQ of 7 have trouble walking on uneven, unpredictable surfaces, patients with a GFAQ of 8 or higher require only minimal support in such an environment. This could partially explain why the movement pattern of some patients are disturbed by the FreeD movement while that of patients in the stable cluster are not. Another explanation might be that actively participating in the mediolateral shift and controlling the trunk movements might require the patients to actively carry their weight. As the median bodyweight support was almost 10% higher than for the stable cluster, this might have been too much. It is possible that a sufficiently low body weight support is a prerequisite for controlled mediolateral trunk and pelvic movements. At the same time, all participants walked with a standardized mediolateral FreeD module excursion of 2 cm per side, which could have been too much for shorter patients. A correlation between height, gait velocity, and mediolateral center of mass displacement has been described before [40]. As the patients in the partially stable cluster tended to be shorter than those in the stable cluster, they might have been more disturbed by the actuated pelvis movements. Therefore, a potential hardware improvement could be a passive guidance of the pelvis that allows the patients to time and modify the magnitude of the movement themselves, instead of an actuated movement that fully guides them, as it currently works. This might induce less disturbance to the patients and could potentially improve the gait pattern of some patients (Table 2).

**Table 2:** Characteristics of the three clusters and the typically developing (TD) children as median and interquartile range in brackets

Cluster	# Patients	Age (y)	Height (cm)	Weight (kg)	Bodyweight Support (%BW)	Speed (km/h)	Timed Up and Go (s)
Unstable	9	11 (3)	142 (18)	26 (27)	23 (13)	1.6 (0.2)	11.4 (7.8)*
Partially stable	15	12 (3)	145 (10)	40 (16)	20 (6)	2 (0.3)	9.7 (7.8)**
Stable	11	14 (2)	159 (15)	52 (16)	11 (5)	2 (0.4)	8 (4.8)
TD	10	11.4 (3)	156 (17)	45 (11.1)	n.a	2.1 (0.2)	~ 5–6 (42)

*Four patients were not able to complete the TUG due to insufficient functional capacity. **Two patients was not able to complete the TUG due to insufficient functional capacity

### Clinical implications

Whether or not to use the FreeD module needs to be decided by the therapist and might depend on the individual therapy goals, skills and deficits of patients. The results presented and discussed above might provide therapists with some of the information necessary for an informed decision. While one drawback of previous studies was that participants of all different severities were included to the analysis, in this study we were able to overcome this problem and analyze differences in behavior between different subpopulations. It is likely that patients exhibiting different gait patterns also show differences in muscle activation patterns [9]. We were able to show that a diverse population reacts individually to guided pelvis movements, and a one size fits all solution does not exist. While some patients behave indeed more physiologically, the gait pattern of others does not profit from the additional pelvis movement. Particularly, patients with unstable trunk kinematics showed less physiological movement patterns than those with stable trunk kinematics comparable to physiological walking. The differences found in trunk control ability between groups suggest that ensuring sufficient trunk control ability before enabling the FreeD module could assist in avoiding compensatory patterns. From a training perspective, the FreeD module might be an interesting option to additionally challenge patients in the partially stable group. As the patients in this group are ambulatory (indicated by the GFAQ Score), they might profit from more natural mediolateral center of mass excursions. However, without additional instructions, as was the case in the present study, the trunk movement can actually worsen. Therefore, therapists should combine the additional kinematic freedom with directed instructions and use the possibility to adapt the magnitude of the excursion and the relative timing of the FreeD module and the leg orthoses [41]. Based on the present findings and the fact that incremental increase in difficulty might be beneficial for motor learning [36], we recommend that therapists should first focus on increasing trunk stability with the disabled FreeD module, before switching it on. This does not mean that a physiological pattern has to be forced at any cost, but if compensatory movements are present, therapists should check if they comply with their therapy goal. Turning on the FreeD module and visually inspecting if the patient can influence the kinematic pattern upon instruction could be a simple way to check whether a patient reacts actively to the additional kinematic freedom.

### Limitations

We want to emphasize that some limitations need to be mentioned. First of all, the current study protocol was a cross-sectional study investigating the relationship between the trunk’s kinematic reactions and clinical assessments. A confirmation of the here established concepts and the implications on the therapeutic setting in a longitudinal study considering the effectiveness is still lacking. We included a wide range of patients. This is a strength because it mirrors the clinical population training with the Lokomat, but at the same time it makes it difficult to establish the real mechanisms, which govern the patient reactions. Complementing such studies with electromyography in the future could help to better understand the mechanisms behind the observed reactions. Furthermore, we did not include the option to individualize the relative timing between the guided pelvis motion and the leg orthoses and the magnitude [41]. The timing and magnitude could also affect whether the patients perceive the pelvis movement as natural or disturbing influencing our study results. Especially for smaller children, 2 cm of excursion could have been too much. The larger amplitudes in the Lokomat than on the treadmill point in that direction. Investigations on how these two parameters affect walking and trunk stability should be performed separately. Secondly, the harness worn in the robot and the application of bodyweight support might restrict the movements of the trunk for some patients. However, the overall range of motion between groups was comparable and did not suggest a significant influence of the harness. At the same time, the body is not rigidly linked to the pelvis support module allowing for some mediolateral pelvis movement even with the FreeD module switched off. This freedom might help to explain why even with a fixed pelvis support, some participants showed pelvis movements similar to treadmill walking. This could indicate that the version of the Lokomat used does not restrict the pelvis movements as much as expected. Furthermore, the current study did also not investigate the influence on leg kinematics. This is important because an enabled FreeD module is usually combined with a lateral sliding of the leg cuffs and therapists should also include the stability of the knee joint in the evaluation of whether or not to use the FreeD module.

## Conclusion

In traditional RAGT with the Lokomat, the pelvis movement is restricted and inhibits physiological weight shifting. Restricting the pelvis has been shown to affect frontal plane kinematics. However, not all patients react similarly to additional actuated pelvis movements. In this study, patients were assigned to three different clusters that showed a distinct movement pattern. Patients with poor trunk control showed an unstable trunk independent of whether the FreeD module was activated or not. Patients with a good trunk control ability showed a stable trunk with the actuated pelvis movement disabled, and either compensatory trunk movements or physiological trunk movements with the actuated pelvis movement enabled. When planning therapy, therapists should consider that some patients are actually disturbed by these movements. Whether the FreeD can lead to long-term improvements is an open question that needs to be evaluated separately.

## Supplementary Information

**Additional file 1: Supplement A.** Standardized Instructions.

**Additional file 2: Supplement B.** Parameter Settings for all participants.

## Data Availability

The datasets supporting the conclusions of this article are available on request and if no ethical reasons prohibit sharing.

## Abbreviations

RAGT: Robot assisted gait therapy
TCMS: Trunk Control Measurement Scale
TUG: Timed up and go test
GFAQ: Gillette Functional Assessment Questionnaire
