# Supplementary material for: Clustering trunk movements of children and adolescents with neurological gait disorders undergoing robot-assisted gait therapy: the functional ability determines if actuated pelvis movements are clinically useful
Source: J Neuroeng Rehabil. 2023 Jun 3;20:71. doi: 10.1186/s12984-023-01200-0 (PMC10239585; doi:10.1186/s12984-023-01200-0)
Supplement: Supplementary file 1 — Additional file 1: Supplement A. Standardized Instructions. [file 12984_2023_1200_MOESM1_ESM.docx]

# Supplemental Material A: Standardized test instructions

**Instruction acclimatization period:**

"You will now walk in the Lokomat for about 10 minutes to familiarize with the Lokomat. Try to walk as independently as possible and actively support the Lokomat."

Corrections phrases during this period (if needed):

"Try to keep your upper body stable without tensing up"

" Put your hands only very loosely on the bars and do not pull from your arms"

"Try to extend your legs well when you stand on them"

"Try to lift your feet well"

"Keep it up! You're doing great"

"Look straight ahead"

**Instruction for all conditions:**

"You will now walk for 3 minutes, during which we will film you. During the 3 minutes we are not allowed to talk to each other and you should look ahead and try to follow the movement of the Lokomat well. Put your hands sideways on the parallel bars, but

support yourself as little as possible."

Corrections phrases during this period (if needed):

- "Try to keep your upper body stable without tensing up"
- "Put your hands only very loosely on the bars and do not pull from your arms"
- "Try to extend your legs well when standing on the bar"
- "Try to lift your feet well"
- "Keep it up! You're doing great"
- "Look straight ahead"
